# Virtual family-centered rounds: a quality improvement initiative to adapt inpatient care during COVID-19 using a human-centred participatory design approach

**DOI:** 10.1186/s12887-023-04091-1

**Published:** 2023-06-13

**Authors:** Melanie Buba, Catherine Dulude, Roisin O’Donnell, Anne Rowan-Legg, Megan Sloan, Matthew Nelson, W. James King

**Affiliations:** 1 Department of Pediatrics, Division of Pediatric Medicine, Children’s Hospital of Eastern Ontario, 401 Smyth Road, Ottawa, ON K1H 8L1 Canada; 2 Children’s Hospital of Eastern Ontario Research Institute, 401 Smyth Road, Ottawa, ON K1H 8L1 Canada; 3 Faculty of Medicine, University of Ottawa, 451 Smyth Road, Ottawa, ON K1H 8M5 Canada

**Keywords:** Virtual care, Virtual rounding, Inpatient telemedicine, Telerounding, COVID-19 pandemic, Family-centered, Infection control, Hospital medicine, Technology implementation, Health systems research

## Abstract

**Background:**

Family-centered rounds (FCR) are fundamental to pediatric inpatient care. During the COVID-19 pandemic, we aimed to design and implement a virtual family-centered rounds (vFCR) process that allowed continuation of inpatient rounds while following physical distancing guidelines and preserving personal protective equipment (PPE).

**Methods:**

A multidisciplinary team developed the vFCR process using a participatory design approach. From April through July 2020, quality improvement methods were used to iteratively evaluate and improve the process. Outcome measures included satisfaction, perceived effectiveness, and perceived usefulness of vFCR. Data were collected via questionnaire distributed to patients, families, staff and medical staff, and analyzed using descriptive statistics and content analysis. Virtual auditors monitored time per patient round and transition time between patients as balancing measures.

**Results:**

Seventy-four percent (51/69) of health care providers surveyed and 79% (26/33) of patients and families were satisfied or very satisfied with vFCR. Eighty eight percent (61/69) of health care providers and 88% (29/33) of patients and families felt vFCR were useful. Audits revealed an average vFCR duration of 8.4 min (SD = 3.9) for a single patient round and transition time between patients averaged 2.9 min (SD = 2.6).

**Conclusion:**

Virtual family-centered rounds are an acceptable alternative to in-person FCR in a pandemic scenario, yielding high levels of stakeholder satisfaction and support. We believe vFCR are a useful method to support inpatient rounds, physical distancing, and preservation of PPE that may also be valuable beyond the pandemic. A rigorous process evaluation of vFCR is underway.

**Supplementary Information:**

The online version contains supplementary material available at 10.1186/s12887-023-04091-1.

## Introduction

Patient and familyinvolvement in health care decisions is recognized as a key component of delivering quality health care [1]. The American Academy of Pediatrics recommends family-centered rounds (FCR) as a fundamental vehicle for involving patients and their families in health care decisions and ensuring patient- and family-centered care [2]. The benefits of FCR include better understanding of the plan of care for patients, families and the health care team, as well as improved communication, satisfaction with care, patient safety and medical trainee education [3]. Traditionally, FCR take place in or just outside a patient’s room and include the patient, family and healthcare providers (defined as physicians, nurses, trainees and any other health care professionals such as dietitians, occupational therapists, physiotherapists, etc.) on the patient’s care team [3]. With the onset of the COVID-19 pandemic, entering patient rooms for FCR was no longer recommended as part of the effort to maintain physical distancing and preserve personal protective equipment (PPE). Therefore, an alternative process was required to ensure that the benefits of FCR could be maintained while adhering to evolving public health recommendations designed to minimize the transmission of SARS-CoV-2, most notably physical distancing. Virtual family-centered rounds (vFCR) have been implemented as an alternative [4–8] to in-person rounds at other centers, but data on outcomes, including stakeholder satisfaction, are lacking [9, 10].

Our aim was to rapidly design and implement a virtual model of FCR that would reduce close contact transmission of SARS-COV-2 and conserve PPE while retaining the benefits associated with FCR during the COVID-19 pandemic. Our process had to adhere to our institutionally-accepted in-person FCR workflow and timing benchmarks, permit multidisciplinary participation, and be acceptable to patients, families, and the multidisciplinary care team [11–14].

## Methods

### Context

The setting was a freestanding academic tertiary care children’s hospital that admits approximately 7000 patients annually; about half of which are managed by the Pediatric Medicine service. Patients with a variety of medical diagnoses—excluding oncology and transplant – are admitted to one of three multidisciplinary acute care teams across four inpatient units. Each team consists of 1 attending staff physician, 1 senior and 2–3 junior residents, 2–3 medical students, 1 clinical pharmacist and 1 bedside nurse. Two teams are geographic, meaning their patients are located on single inpatient unit, and the third has patients distributed across four inpatient units. The nurse-to-patient ratio is generally 1:4 but may vary depending on patient care needs/acuity.

In-person FCR were instituted in 2014 and the standard content, chronology and roles were most recently updated in 2019 to reflect evolving FCR best practices [15]. The hospital uses an integrated electronic health record (Epic Systems Corp., Aug 2019) with a combination of fixed and mobile devices supporting access to patient charts. At the outset of the COVID-19 pandemic, a privacy legislation-compliant video conferencing platform was made available organizationally to facilitate virtual care. Hospital executive leadership supported the vFCR project and provided in-kind donations of human resources, information technology (IT) infrastructure, hardware and software.

This project was deemed to be quality improvement and exempted from full review by our institutional research ethics board (REB) the Children’s Hospital of Eastern Ontario IRB #1, IRB00002747.

### Approach

We opted to follow a human-centred participatory design approach, which emphasizes understanding the needs and experiences of people using or being impacted by the problem or solution and places key stakeholders and knowledge-users at the center of the project design and implementation [16, 17]. Accordingly, a multidisciplinary project team was formed including quality improvement specialists, a human factors engineer, front-line physicians, nurses, a resident and family of previously admitted patients. The majority of project team members had prolonged engagement with the inpatient medicine environment, and with FCR. The vFCR process was initially developed by a physician and human factors engineer in consultation with staff and medical staff and using available software, hardware and IT infrastructure: privacy legislation-compliant videoconferencing software (Zoom Video Communications, San Jose, CA), tablets with ear buds (Apple Inc., Cupertino, CA) and laptop computers. Minimal cost was incurred to purchase protective tablet covers that enabled frequent disinfection. Iterative audit-and-feedback improvement cycles were used to refine and improve the vFCR content and process [18].

### The virtual family-centered rounds process

On admission, patients and families received an information sheet about vFCR. A rounding schedule was created daily for each inpatient unit by the unit clerk based on nursing assignments. All patients and families were invited to participate in vFCR by their nurse during their morning assessment. The unit clerk joined the vFCR videoconference with two tablets: one for the bedside nurse and one for the patient and family—and plugged-in headsets to each. The display names reflected the inpatient unit and role of the user (e.g. 5E Nurse). The tablets were set on a rolling table with hospital grade cleaning wipes (Oxivir Tb Disinfectant Wipes) [13, 14].

Patient handover for physician teams occurred at 0730. Patients were assigned to medical trainees and seen in-person prior to rounds, if possible. Medical trainees joined virtual family-centered rounds from conference rooms where they were physically distanced and wearing PPE (i.e. universal masking as per hospital policy), while attending physicians and pharmacists used their personal offices and joined virtual rounds using laptop computers or mobile devices. The attending physician or senior resident (i.e., meeting host) started the videoconference and managed participant entry via the virtual waiting room. All participants connected with video on, and their names and roles clearly displayed (e.g., Dr. Smith—Resident).

The rounding team began vFCR at a prescheduled time (e.g., 0930). The first nurse provided the first patient/family with a tablet and headset, and then moved to a quiet and private location for the duration of their patient’s round. The meeting host facilitated rounds following the FCR standard content (see Additional File 1). When rounding for that patient was complete, the nurse cleaned the tablet and headsets and moved to the next scheduled patient or handed off the equipment to the next nurse on the rounding schedule. At the end of vFCR the tablets were returned to the unit clerk for charging and safe storage [13, 14].

### Studying virtual family-centered rounds

This project took place from April 6 to July 31, 2020. During this period the average patient census was 11 patients per team (SD = 3.7). During the project period, our patient census was approximately 35% lower than typical for the same time of year, which we attribute to the COVID-19 pandemic. Outcome measures included stakeholder satisfaction, perceived effectiveness and perceived usefulness of vFCR. To measure effectiveness, we asked patients and families if they felt like valued partners in their (child’s) care during rounds. To measure usefulness, we asked stakeholders how well they understood their/their patients’ care plan. Our process measure was technology ease of use. Balancing measures were time per patient round and transition time between patients.

Data collection tools were developed for vFCR auditors (off-duty front-line nurses) who observed rounds virtually. Auditors were trained on the audit tool (see Additional File 2) and met as a group after initial audits to review data, engage in peer debriefing and ensure consistent data collection. Auditors also gathered qualitative feedback from nurses and conducted semi-structured interviews with patients and families post-rounds. Questionnaires (see Additional File 3) used a 5-point Likert scale and free text responses, and were distributed to members of the care team, patients ≥ 12 years, and families. Rounds were initially audited daily for timing, adherence to standard content and process. Patient and family interviews were conducted by nurse auditors based on resource availability. The data from audits and interviews were reviewed twice weekly by the multidisciplinary project team and changes were made to improve the vFCR process based on data and feedback. As the process stabilized, auditing was gradually reduced to once per care team per week.

### Data analysis

Audit data were used to generate X-bar S control charts of rounding and transition times. Data were reviewed for quality by 2 members of the project team (CD, MN) and 125 data points were corrected based on information provided in auditor notes. A goal of 10 min per patient round was determined using data from previous quality improvement work on FCR at our centre combined with a literature review [19, 20]. A 4-min maximum transition time between patients was targeted for efficiency and buy-in from physicians. Quantitative analysis of questionnaire data identified participants’ level of satisfaction and perceptions of vFCR effectiveness and usefulness. Content analysis of qualitative questionnaire and interview data helped develop a better understanding of the perceived value and barriers to a sustainable operational model.

## Results

The iterative development and implementation of vFCR fell into three phases: 1. Initial process development & rapid improvement (April–May), 2. Process standardization & monitoring (May–June), and 3. Transition to operations (July); these are summarized in Table 1.

**Table 1:** Development and implementation of virtual family-centered rounds

	**Plan ➜ **	**Implement ➜**	**Study ➜**	**Adjust ➜**
1. Initial process development	• Develop initial vFCR standard process and technical setup	• Training and go-live of vFCR • Daily audits of vFCR (quantitative measures of time and qualitative feedback), semi-structured interviews with families and care team members • Bi-weekly multidisciplinary project team meetings	• Analysis of quantitative and qualitative data and trends	• Adjusted technical setup to support sound quality ◦ Added second tablet and headsets (one for Patient/family, one for nurse) ◦ USB conference speakers (Jabbra Speak510) for each conference room to eliminate feedback—one laptop serves as speaker and microphone for room • Update vFCR standard process to clarify roles and responsibilities, technical setup • Developed vFCR standard process tip sheets for health care providers focused on roles and responsibilities • Posted rounding schedule at nursing station • Standardized tech setup for nursing including: a rolling height-adjustable table, two tablets and headsets, wipeable tips sheets, cleaning supplies
2. Process standardization	• Developed feedback questionnaires for all stakeholder groups • Developed standard process for pharmacy attendance at rounds • Scheduled weekly check-in with nursing leadership and project team	• Continue bi-weekly audits for each care team • Disseminate feedback questionnaires (in-person and electronic)	• Analyse data from audits and questionnaires	• Delivered training on vFCR facilitation for physician team members • Stopped sharing of rounding tip sheet during videoconference so patient/family sees bigger faces on screen • Weekend rounds changed back to in-person due to lack of support staff and small team size • Upgraded wireless access points on the units to improve reliability and optimized videoconference settings to save steps (e.g., auto-join internet audio) • Developed downtime procedures
3. Transition to operations	• Developed operational feedback loop involving daily check-ins between nursing and physician team leads	• Communicated vFCR process and outcome measures, key actions that support efficient and effective rounds, downtime procedures, operational metrics and support structure • Posted vFCR standard process and tip sheets on intranet	• Periodic audits of rounding and transition times, weekly check-ins with nursing leadership and to ensure process being followed	• Reinforcement of process and downtime procedures as needed • Project oversight transitioned to Inpatient Patient Care Leadership Team

*vFCR* Virtual family-centered rounds

Two hundred and forty health care providers participated in vFCR during the 4-month project period. One thousand seven hundred and ninety-two individual vFCR were audited for adherence to vFCR standard content and technology issues. These audits spanned an estimated 471 patient admissions.

### Outcome measures

Of the 240 health care providers who participated in vFCR during the project period, 28.7% (69/240) completed feedback questionnaires (nurse (33, 47.8%), physician trainee (20, 29.0%), staff physician (16, 23.2%), healthcare professional (0)). Of these, 74% (51/69) were satisfied or very satisfied with the process, and 88% (61/69) agreed or strongly agreed they had a good understanding of their patients’ care plan after rounds [13]. We tracked family participation across 1459 vFCR and found that family was present during 46% (*n*= 674) of rounds. Patient and family feedback questionnaires were distributed on 6 individual days between May 29 and July 6 and we obtained 33 responses. Of these, 79% (26/33) of respondents indicated they were satisfied or very satisfied with vFCR, 88% (29/33) agreed or strongly agreed they felt like a valued partner in their child’s care during rounds, and 88% (29/33) agreed or strongly agreed the team used language they understood and the plan of care was clear [13]. Forty-three patients and family members were interviewed post-rounds on 7 separate days between April 29 and May 28; analysis revealed 65 positive comments, 5 neutral comments, 36 negative comments and 8 improvement ideas across 7 themes (Table 2). Notably, 3 family members who had experience with in-person FCR indicated they preferred vFCR [13].

**Table 2:** Patient and family interview data themes and categories with representative codes

**Themes**	**Categories**			
	**Positive**	**Neutral**	**Negative**	**Idea**
**Overall experience**	• Less intimidating than in-person rounds • Felt part of the team • Value having the whole team together • Adapting care in a way that includes families		• Patient unable to participate because parent had headset	
**Tech setup**	• Headset better than no headset • No tech issues • Good sound quality		• One tablet/headset does not support patient and parent participation • Remote family can’t participate • Background noise makes it hard to hear (not using headset)	• Bigger screen • Do not auto-mute • Add headset/splitter
**Process**	• Consistent process daily • Nurse managed tech for them		• Team roles not clear • Parent/patient role unclear • Unclear who should speak next	• Standardize introductions • Display role on screen
**Infection Control**	• Fewer people in/out of patient room	• Prefer in-person but understand why virtual important during pandemic	• Sharing headsets unclean	
**Staff comfort/skill**			• Unclear who should speak next • Staff did not adhere to standard content • Nurse not comfortable with tech	• Standardize content/process
**Privacy**	• More private than in-person rounds • Roommate could not hear			
**Language**			• Required translation	

### Process measure

At the beginning of the project auditors regularly reported sound and connection reliability issues, but as the project team responded with changes to process, infrastructure and end user device setup, technology reliability improved. For example, virtual meeting connection reliability was optimized with wireless network settings and the addition of high-capacity wireless access points (re-purposed from unused meeting rooms). Eighty-two percent (27/33) of patients and families who responded to the questionnaire agreed or strongly agreed that the technology used for vFCR worked well and was easy to use.

While not directly measured, auditor observations revealed care team adherence to FCR standard content improved over the course of the project. However, patient and family feedback suggest additional room for improvement: for example, only 58% (19/33) of questionnaire respondents agreed or strongly agreed that everyone participating in vFCR introduced themselves and that participants’ roles were clear.

### Balancing measures

**Fig. 1:**
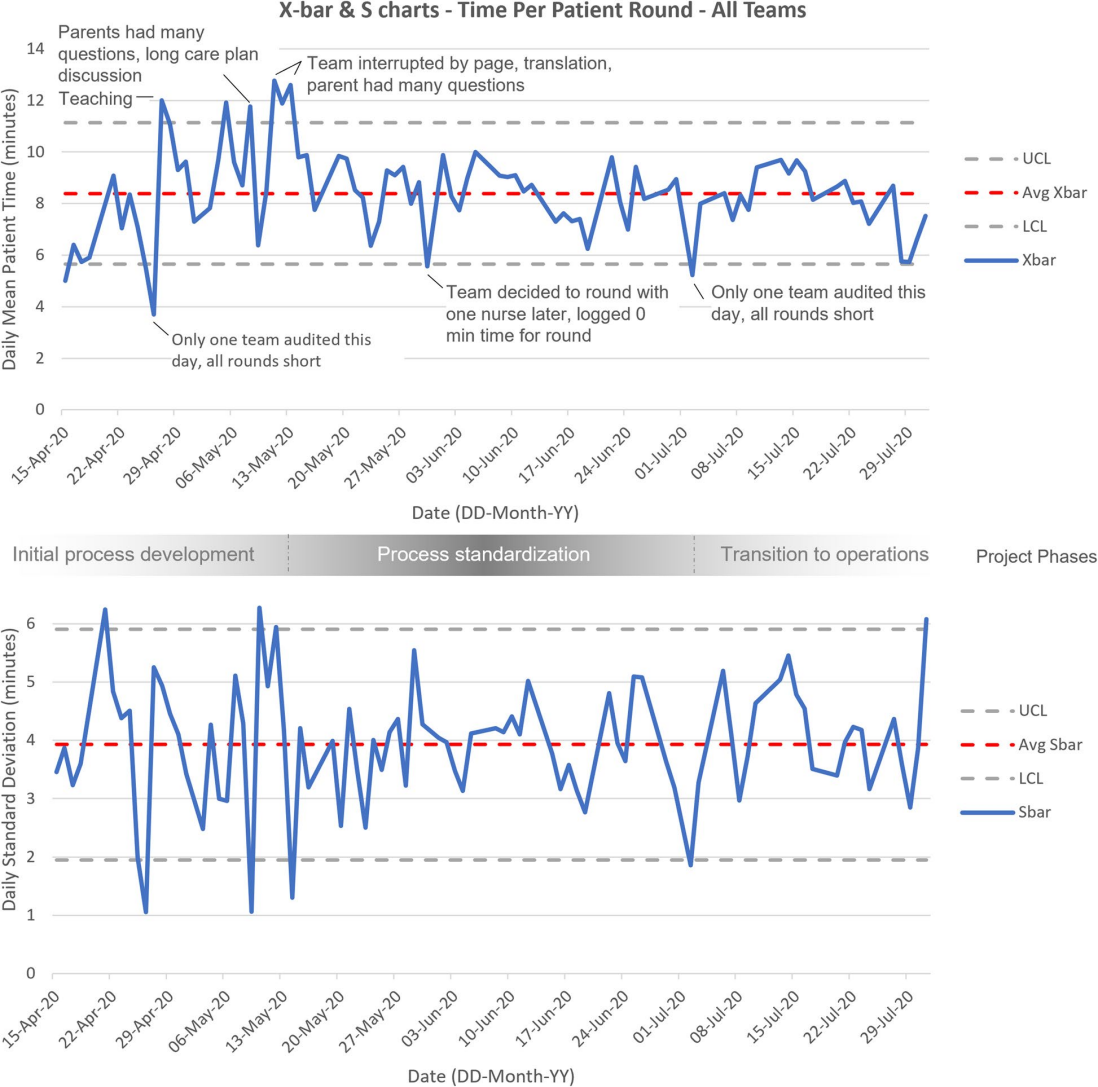
X-bar S control charts for time per patient round. Top, X-bar Chart where Avg X-bar is the average mean time per patient round. Bottom, S chart, where Avg S-bar is the average standard deviation of rounding times. UCL is the upper control limit, LCL is the lower control limit. Data points outside control limits represent cases of variation and analysis of audit data allowed for identification of special cause variation (SCV) in most cases

**Fig. 2:**
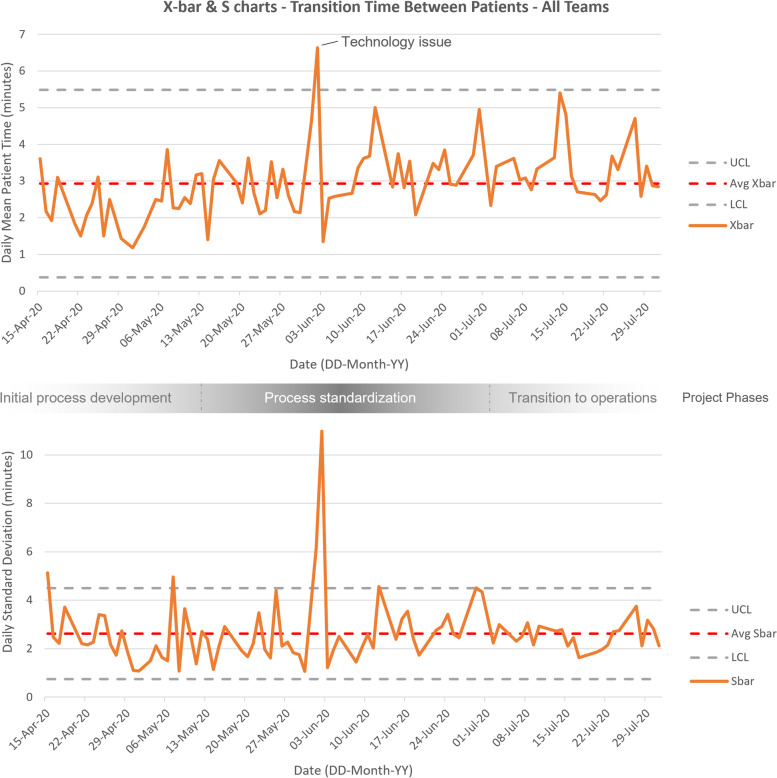
X-bar S control charts for transition time between patient rounds. Top, X-bar chart where Avg X-bar is the average mean transition time between patient rounds. Bottom, S chart, where Avg S-bar is the average standard deviation of transition times. UCL is the upper control limit, LCL is the lower control limit. Data points outside control limits in later phases represent cases of variation attributed to special cause (SCV) identified through analysis of audit data

Analysis of 1792 vFCR revealed the average time to round on a patient was 8.4 min (SD = 3.9; Fig. 1) and the transition time between patients averaged 2.9 min (SD = 2.6; Fig. 2) [13]. The X-bar S charts generated for these data show rounding and transition times initially varied greatly. Special cause variation represented cases of nurse absence, translation needs, technology issues, physician team interruption and rounding on complex patients. During the project period, the overall efficiency of vFCR improved, as evidenced by a reduction in timing variation.

We did not explicitly include patient safety as a balancing measure. However, safety events reported to our institutional safety reporting system did not show a statistically significant change in reporting on the medical inpatient units where vFCR occurred compared with the overall hospital rate (Fisher’s exact test; *P* = 0.4249). Also, no patient safety incidents related to vFCR were brought to the attention of the project team or inpatient leadership.

## Discussion

The COVID-19 pandemic has changed many aspects of care delivery and highlighted that in-person care may not always be ideal when attempting to limit the transmission of infectious agents. With the move towards virtual care, we should continue to prioritize family-centred approaches to maintain safe and high quality care [1]. This project demonstrates the ability to conduct FCR through a virtual platform and suggests vFCR are an acceptable alternative to in-person FCR in terms of stakeholder satisfaction, perceived effectiveness and perceived usefulness in these unique times.

While other pediatric centres have trialled vFCR [4–8], our process appears to align more closely to FCR best practices in terms of content, scheduling and role clarity [15, 19, 21]. Importantly, our process led to optimizations in timing and overall efficiency; well-known barriers to implementation of successful FCR [3]. A virtual rounding process developed in the neonatal intensive care setting reported similar benefits including improved efficiency, shorter rounding times, fewer interruptions, increased safety, and improved physical distancing [7]. This evidence of similar processes successfully implemented in different care contexts highlights the utility and adaptability of the virtual rounding approach.

A robust wireless network and access to video-capable tablets with headsets allowed for rapid implementation of our vFCR process. Because vFCR is dependent on technology and technology options vary across organizations, identification of usability requirements and technical factors that would maximize user acceptance [22, 23] and facilitate implementation and adoption would be important considerations for other organizations implementing a similar process.

Rounding and transition time targets were achieved and were important contributors to securing stakeholder buy-in. Unanticipated benefits of vFCR included increased confidentiality and environmental noise reduction as patients, families and nurses all wore headsets. Furthermore, reducing the number of providers at the bedside was perceived by some patients and families as safer and less intimidating, which is consistent with the literature [13, 24].

### Lessons learned

To realize the full potential of vFCR, adequate space is required for physicians and trainees to join virtual meetings unmasked. Limited availability of private office space at our hospital meant medical trainees had to remain masked during vFCR, which hindered their ability to maintain non-verbal communication (e.g. understanding and/or support communicated through facial expression), which is particularly important when conveying empathy and compassion during difficult and sensitive conversations [7]. To this end, we believe the overall experience with vFCR and perceptions of rapport would have been improved if physician teams could have joined videoconferences unmasked.

In addition, we recognized health care team training on the vFCR process and technology was essential and should be prioritized. While the structure and content of vFCR remained the same as in-person FCR, learning to use vFCR technology was an additional challenge. Moreover, where in-person rounds enabled the use of non-verbal cues (e.g. body position and movement) to support communication, virtual interactions require explicit verbal commands, for example, “…moving on to the next patient”. To support our teams, educational sessions and tip sheets were created, and timely, in-person support from the project team was available.

Similar to other studies [20, 25], the creation of a rounding schedule was essential to ensure availability of bedside nurses for vFCR. Given that implementing and adhering to a rounding schedule was a change from our usual in-person process, we initially instituted a simple 10-min-per-patient schedule for vFCR. We later modified the scheduling system to allow care teams to request longer or shorter rounding times based on predicted patient needs. Unfortunately, we were not resourced to have a rounds coordinator, which has been found to enhance the timeliness of a scheduled rounding process [20].

Finally, the ability for patients and families to participate in vFCR is essential. The tablets we used did not support a headset splitter, necessitating removal of the headset so both patient and family could hear. This occasionally impacted privacy and sound quality. Additionally, using a continuous videoconference meeting for all patients facilitated provider workflow but prevented us from inviting offsite family members to join vFCR for security reasons. While the process we developed to allow offsite family members to join by phone was appreciated, ideally, we would have been able to offer participation by videoconference.

### Limitations

While the simple project design and iterative improvement process yielded clear results in terms of stakeholder perceptions of vFCR, there was limited evaluation of satisfaction and effectiveness of in-person FCR at our centre and a lack of reliable baseline data against which we could compare. Additionally, certain data were not rigorously captured, which limited our ability to substantiate important observations. For example, staff physicians estimated 90% nursing attendance during vFCR compared to 65% for in-person FCR, however, this was not explicitly tracked by auditors and therefore could not be included in results. The low patient/caregiver sample (*n* = 33) for questionnaire responses is another limitation that could suggest self-selection bias. However, questionnaire responses were corroborated by interviews and qualitative feedback collected by auditors. We attribute the low number of questionnaire responses to low (46%) family participation in vFCR, which may have been, in part, due to COVID-19-related visitor restrictions. In addition, there were only 6 days of questionnaire distribution due to resource limitations, further reducing the potential number of respondents.

While we did not formally analyze cost-effectiveness of the intervention, there were minimal costs associated with the switch from in-person to virtual rounds given our use of existing technology and hardware. Additionally, by adopting a virtual rounding process, we substantially reduced the demand for PPE and estimate PPE cost savings of approximately $36,000 CAD per month ($6.79 CAD per person × 8 members per care team × 11 patients per day × 3 rounding teams × 20 days per month) [13].

Finally, this project was performed within a single academic tertiary care centre undergoing rapid change in response to the COVID-19 pandemic. The learnings, therefore, may not be generalizable to all contexts.

## Conclusion

We rapidly developed and implemented vFCR for patients admitted to the Pediatric Medicine service without the need for significant investment in technology or additional human resources. Our evaluation revealed vFCR to be an acceptable, effective and useful alternative to in-person FCR, yielding high levels of stakeholder satisfaction and support. We believe vFCR are a valuable way to support inpatient rounds, physical distancing and preservation of PPE. While there has recently been a return to in-person FCR, our findings suggest vFCR may be beneficial for isolated patients by facilitating safe, effective and inclusive FCR in both unique and usual times. A rigorous process evaluation of vFCR was conducted in 2021 and an analysis of findings is currently underway.

## Supplementary Information

**Additional file 1. **Family-Centred Rounds Standard Content.

**Additional file 2. **Virtual Rounds Audit Tool.

**Additional file 3. **Virtual Family Centred Rounds Feedback Questionnaire for health care team members.

## Data Availability

The dataset used and/or analyzed during this project are available from the corresponding author on reasonable request.

## Abbreviations

FCR: Family-centered rounds
PPE: Personal protective equipment
vFCR: Virtual family-centered rounds
REB: Research ethics board
IT: Information technology
PHIPA: Personal Health Information Protection Act
SD: Standard deviation
CAD: Canadian dollar
