# Supplementary material for: Virtual family-centered rounds: a quality improvement initiative to adapt inpatient care during COVID-19 using a human-centred participatory design approach
Source: BMC Pediatr. 2023 Jun 13;23:289. doi: 10.1186/s12887-023-04091-1 (PMC10261833; doi:10.1186/s12887-023-04091-1)
Supplement: Supplementary file 1 — Additional file 1. Family-Centred Rounds Standard Content. [file 12887_2023_4091_MOESM1_ESM.pdf]

Family-Centered Rounds Standard Content	
**Step:**	**Actions:**
1. Introduction	Care team members introduce themselves and their role.
2. Initial report	Most Responsible Learner (MRL) shares:<br>• Patient identification<br>• Prioritized Problem List
3. Nursing report	1. Recent vital signs, especially abnormal vital signs from last 24h<br>2. Recently administered medications (especially as-needed meds)<br>3. Weight, weight changes<br>4. Intravenous fluids and/or diet/feeds: Type, rate, reason<br>5. For NG – type, date of insertion, external length<br>6. Ins & Outs over the last 6 and 24h: Urine, stool, emesis, all drains & lines<br>7. Isolation Status<br>8. Current Monitoring (i.e. continuous cardiorespiratory monitoring, nurse in room status, etc.)<br>9. Scheduled tests for the next 24 hours<br>10. Nursing Barriers to Discharge<br>11. Concerns about patient and/or plan of care
4. Medication review	Pharmacy or physician team member
5. Investigation Review	MRL shares:<br>• Physical exam<br>• Lab results<br>• Imaging results<br>• Consults
6. Health Care Professional	Provides a report if applicable
7. Care plan	MRL will:<br>• Prompt patient and family for input related to care plan to encourage shared decision making<br>• Impression/Summary statement<br>• Plan of care (focus on the next 24h)<br>• Criteria and barriers for discharge<br>• Estimated discharge date (EDD)
8. Order read back	Any new orders are read back.
9. Patient and Family summary	Senior resident: lay terms, in primary/first language (if possible)<br>Ask for and answer any questions from patient/family

Taken from: https://www.cheo.on.ca/en/resources-and-support/vfcr-toolkit.aspx#Family-Centred-Rounds-Standard-Content
