# Supplementary material for: Virtual family-centered rounds: a quality improvement initiative to adapt inpatient care during COVID-19 using a human-centred participatory design approach
Source: BMC Pediatr. 2023 Jun 13;23:289. doi: 10.1186/s12887-023-04091-1 (PMC10261833; doi:10.1186/s12887-023-04091-1)
Supplement: Supplementary file 3 — Additional file 3. Virtual Family Centred Rounds Feedback Questionnaire for health care team members. [file 12887_2023_4091_MOESM3_ESM.pdf]

Virtual Family-Centred Rounds

This survey will take less than 5 minutes to complete. The information you share is anonymous and will be used to improve virtual family-centred rounds.

Please indicate your role

- ◯ Nursing
- ◯ Medical Learner (Resident, Student)
- ◯ Staff Physician
- ◯ Health Care Professional
- ◯ Other

Please indicate your unit/team

- ◯ 4E/5E
- ◯ 4W
- ◯ 4N
- ◯ Float
- ◯ Red Team
- ◯ Purple Team
- ◯ Bronze Team
- ◯ Other

What do you like about the new virtual family-centred rounds process?

____

What is challenging about the new virtual family-centred rounds process?

____

What would make virtual family-centred rounds more valuable for you?

____

How satisfied are you overall with virtual family-centred rounds?

- ◯ Very dissatisfied
- ◯ Dissatisfied
- ◯ Neutral
- ◯ Satisfied
- ◯ Very satisfied

After virtual family-centred rounds, do you feel you have a good understanding of your patient's care plan for the day?

- ◯ Completely disagree
- ◯ Disagree
- ◯ Neutral
- ◯ Agree
- ◯ Completely agree

Do you feel that virtual family-centred rounds are an essential part of patient care and should be prioritized?

- ◯ Completely disagree
- ◯ Disagree
- ◯ Neutral
- ◯ Agree
- ◯ Completely agree

Do you have any additional comments about virtual family-centred rounds?

____
